# Suppression of glutathione system and upregulation of caspase 3-dependent apoptosis mediate rohypnol-induced gastric injury

**DOI:** 10.1080/13510002.2022.2074128

**Published:** 2022-05-10

**Authors:** R. E. Akhigbe, D. T. Oluwole, T. E. Adegoke, M. A. Hamed, D. C. Anyogu, A. F. Ajayi

**Affiliations:** a Reproductive Physiology and Bioinformatics Research Unit, Department of Physiology, Ladoke Akintola University of Technology, Ogbomoso, Nigeria; b Reproductive Biology and Toxicology Research Laboratories, Oasis of Grace Hospital, Osogbo, Nigeria; c Department of Physiology, Joseph Ayo Babalola University, Arakeji, Nigeria; d Brainwill Laboratories and Biomedical Services, Osogbo, Nigeria; e Department of Veterinary Pathology and Microbiology, University of Nigeria, Nsukka, Nigeria

**Keywords:** Rohypnol, benzodiazepines, drug abuse‌, stomach, COX, glutathione‌, oxidative stress, inflammation, apoptosis, caspase 3

## Abstract

**Objectives:** This study investigated the impact of rohypnol on gastric tissue integrity.

**Methods:** Forty male Wistar rats were randomized into control, low dose rohypnol-treated, high dose rohypnol-treated, low dose rohypnol-treated recovery and high dose rohypnol-treated recovery groups.

**Results:** Rohypnol caused significant rise in gastric malondialdehyde (MDA), oxidized glutathione (GSSG), nitric oxide (NO), tumour necrotic factor-α (TNF-α), and interleukin-6 (IL-6) levels. Also, rohypnol caused reductions in gastric reduced glutathione (GSH) (as well as GSH/GSSG), and activities of superoxide dismutase (SOD), catalase, glutathione-S-transferase (GST), glutathione peroxidase (GPx), cyclo-oxygenase (COX-2). Furthermore, rohypnol upregulated caspase 3 activity and induced gastric DNA damage, evident by a rise in 8-hydroxydeoxyguanosine (8-OHdG) and DNA fragmentation index (DFI) in gastric tissue. These alterations were coupled with reduced gastric weight and distorted gastric cytoarchitecture. Cessation of rohypnol caused a significant but not complete reversal of rohypnol-induced gastric damage.

**Conclusion:** This study revealed that rohypnol induced gastric injury by suppressing glutathione content and COX-2 activity, and upregulating caspase 3-dependent apoptosis, which was partly reversed by rohypnol withdrawal.

## 1. Introduction

Drug abuse is a global threat and the attendant adverse effects are associated with socio-medical challenges. Several drugs such as opioids, cocaine, cannabis, amphetamine and benzodiazepines have been identified as common substances of abuse [1]. According to a study conducted in Nigeria, the most common substances of abuse are cannabis, opioids (such as codeine and tramadol), and benzodiazepines (such as diazepam and rohypnol) [2].

Rohypnol is a potent hypnotic and pre-anesthetic agent [3]. It is a sedative drug prescribed for the short-term treatment of insomnia [4,5]. Rohypnol may induce aggression and excitability by reducing the respiratory motor amplitude and frequency through increased γ-amino butyric acid (GABA) activity [6]. It has also been associated with poor mental state, impaired muscle control and psychomotor skills, organ toxicity [7], suppression of hypothalamic-pituitary-testicular axis [8], and rarely death [9]. Despite the prevalence of rohypnol abuse and its possible organ damage, including gastric injury, studies reporting the effects of rohypnol exposure on gastric tissue are scarce.

Glutathione (GSH), a tripeptide (glutamate, cysteine and glycine), has been shown to have multiple functions including antioxidant effects [10]. The glutathione system detoxifies reactive oxygen and nitrogen species (ROS/RNS), and electrophiles generated in the body [11]. Aside acting as an essential cellular redox buffer to promote optimal regulation of a range of redox-sensitive biochemical processes [12], it also modifies the activity of several proteins and intracellular signaling molecules while inhibiting the activity of some enzymes [11]. In addition, the system is crucial in regulating the expression of pro-inflammatory cytokines such as tumor necrosis factor-α (TNFα), interleukin (IL)-1β and IL6 [11]. Furthermore, the glutathione system, through translocation of nuclear factor erythroid 2-related factor (Nrf2) into the nucleus, maintains cellular stability and confers protection against DNA damage and apoptosis [13–15].

Caspases are a family of cysteinyl aspartate-specific proteases and a central regulator of apoptosis and DNA fragmentation [16]. The activation of caspase 3, an executioner caspase, is the key event in cell apoptosis [17] via the intrinsic pathway involving the mitochondrion acting as an intracellular death receptor with the release of cytochrome *c* and induction of apoptosome formation or the extrinsic pathway, which activates caspase 3 directly [18]. It is well documented that oxidative stress and inflammation are important factors that regulate apoptosis signal-regulating kinase [19] and promote apoptosis through activation of caspase 3 [20].

Given the aforementioned information, this study evaluates the effect of rohypnol on gastric integrity and redox state. In addition, since the glutathione system and caspase 3-mediated apoptosis play contrary roles in cell stability via oxidative-sensitive signaling, which has been implicated in various tissue/organ damage [13,14,21], the roles of glutathione system and caspase 3 activity in rohypnol-induced alteration in gastric redox state were probed.

## 2. Methodology

### 2.1. Animals and treatment

Forty (40) male Wistar rats weighing 180–200 g were kept under natural conditions and allowed free access to standard rat chow and water. The rats were acclimatized for 14 days and then randomized into five (5) groups (*n* = 8). The control group received 1 ml of distilled water, the low-dose rohypnol-treated group received 2 mg/kg body weight of rohypnol, the high-dose rohypnol-treated group had 4 mg/kg body weight of rohypnol, and the low- and high-dose rohypnol-treated recovery groups were treated with 2 mg/kg and 4 mg/kg of rohypnol respectively and immediately allowed some recovery period during which no drug was administered. Administration was once daily via gavage for four weeks and the recovery group was allowed another four weeks of drug-free period. The animals were weighed daily, and the dose administered was adjusted per the present weight. The dose and administration route are as seen in humans and as earlier documented [8]. The dose of 2 mg/kg is the adult human equivalent dose, while the 4 mg/kg was obtained as the submaximal peak dose from the dose–response curve in our pilot study as earlier reported [22]. The current study was carried out in accordance with the guidelines of the National Institute of Health using the guide for the care and handling of laboratory animals (NIH Publication No. 80-23; revised 1978) and International Guiding Principles for Biomedical Research. The Ethics Committee of Oyo State Ministry of Health, Ibadan, approved the experimental protocol.

### 2.2. Sample collection

A day after the last dose, the rats were fasted overnight, weighed, and sacrificed under intraperitoneal ketamine/xylazine anesthesia as earlier reported [23]. The stomach was immediately harvested and the gastric contents were extruded. The stomach was then opened along its greater curvature, washed, weighed and recorded as absolute gastric weight. The initial body weight of each rat was subtracted from the final body weight to obtain the body weight gain. The percentage of the ratio of the gastric weight to final body weight was obtained and recorded as relative gastric weight [24]. The gastric mucosa was examined under stereomicroscope with 10× magnification, and the gastric injury lesion index was determined using Guth grade standard as previously reported [25]. Spot erosion grade 1; the length of erosion less than 1 mm grade 2; 1–2 mm length of erosion grade 3; 2–3mm length of erosion grade 4, more than 4 mm length of erosion grade 5; and the width more than 1 mm is multiplied by 2. Scoring was done by two experts who were blinded to the study protocol. The mean value from the two experts was used as the score for each animal. Using a glass homogenizer, the stomach was homogenized in an appropriate volume of cold phosphate buffer solution, and the homogenate was centrifuged at 10,000 g for 15 min at 4°C to get the supernatant for biochemical assay.

### 2.3. Biochemical assay

#### 2.3.1. Evaluation of markers of oxidative stress and antioxidants systems

The concentrations of malondialdehyde (MDA), reduced glutathione (GSH), and oxidized glutathione (GSSG) in the gastric tissue were determined by colorimetric method as earlier reported [15,19,26]. Also, the gastric activities of superoxide dismutase (SOD), catalase, glutathione peroxidase (GPx), and glutathione-S-transferase (GST) were determined by colorimetric method as earlier documented [15,19,27].

#### 2.3.2. Evaluation of markers of inflammation

Nitric oxide (NO) level in the gastric tissue was assayed using Griess reaction as earlier described [28,29], while gastric levels of tumor necrotic factor-α (TNF-α) and interleukin-6 (IL-6), and cyclo-oxygenase-2 (COX-2) activity was assayed using ELISA kit (Elabscience Biotechnology Co., Ltd, USA) following the manufacturer’s guideline.

#### 2.3.3. Evaluation of markers of genotoxicity, DNA fragmentation, and apoptosis

Gastric concentration of 8-hydroxydeoxyguanosine (8-OHdG), and gastric DNA fragmentation index (DFI) and caspase 3 activity were used as markers of genotoxicity (DNA integrity) and apoptosis respectively. Gastric 8-OHdG was measured using the commercially available ELISA kit (Elabscience Biotechnology Co., Ltd) per the manufacturer’s guideline. Gastric DFI was determined using diphenylamine (DPA) methods [30,31]. Gastric caspase 3 activity was assayed using ELISA kits (Elabscience Biotechnology Co., Ltd) following the manufacturer’s guideline.

### 2.4. Histopathological studies

A section of the gastric tissues was fixed in 10% neutral-buffered formalin (v/v) and embedded in paraffin wax. Sections of about 5–6 µm thickness were obtained and stained with hematoxylin and eosin (H&E) for histopathological examinations. Sections were examined with light microscopy and photomicrographs were taken at ×400 magnification. Histopathological examination was done by two experts who were blinded to the study protocol at ×100 and ×400 magnification. Gastric mucosa pathological injury Integrals were calculated according to the scoring standard reported by Masuda et al. [32], namely, 0 point for normal tissue, 1 point for epithelial damage of surface layer, 2 points for hyperemia or edema of the upper stratum, 3 points for hyperemia, edema or even hemorrhage of stratum medium or under layer. Upper mucosal glands with structural disorders or necrosis had a score of 4 points, and the tissue with deep ulcer or necrosis had a score of 5 points. Scoring was done by two experts who were blinded to the study protocol. The mean value from the two experts was used as the score for each animal.

### 2.5. Statistical analysis

Statistical analysis was conducted using Graph Pad Prism for Windows (Version 7). D’Agostino Pearson Omnibus and Shapiro–Wilk normality tests were conducted to ascertain that the data set were normally distributed. One-way analysis of variance (ANOVA) followed by Tukey’s *post hoc* test was used to test for significance across and between groups. Data are presented as mean ± SD. Values of *P* < .05 were accepted as statistically significant.

### 3. Results

### 3.1. Effect of rohypnol on body weight gain and gastric weight

There was no significant change in the body weight gain across the groups. Also, high dose rohypnol treatment caused a significant reduction in the absolute gastric weight. The observed high dose rohypnol-induced reduction in absolute gastric weight was however reversible. Similarly, high dose rohypnol treatment caused a significant but reversible reduction in the relative gastric weight (Table 1).

**Table 1.** Effect of rohypnol on body weight gain and relative gastric weight.

Group/parameter	Control	Low-dose rohypnol	High-dose rohypnol	Low dose- rohypnol recovery	High dose- rohypnol recovery
Body weight gain (g)	46.50 ± 3.52	48.25 ± 3.61	44.50 ± 3.34	51.00 ± 3.50	47.50 ± 3.14
Absolute stomach weight (g)	14.6 ± 1.80	12.7 ± 1.33	11.7±1.73*	14.8 ± 1.80^∼^	13.4 ± 1.93
Relative stomach weight (%)	6.15 ± 0.76	5.34 ± 0.880	4.95 ± 0.69*	6.11 ± 0.89	5.39 ± 0.92

Notes: Values are presented as mean ± SD. *P < .05 vs control; ^#^*P* < .05 vs low-dose; ^∼^*P* < .05 vs high-dose.

### 3.2. Effect of rohypnol on markers of oxidative stress

**Figure 1.**
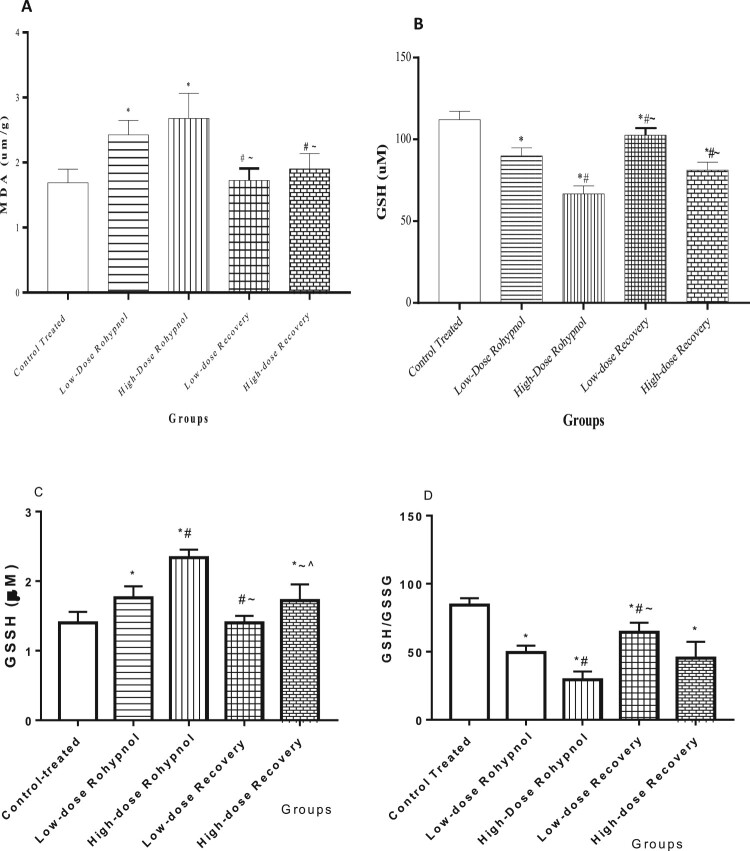
Effect of rohypnol on gastric levels of malondialdehyde, MDA (A), reduced glutathione, GSH (B), oxidized glutathione (C), and GSH/GSSG ratio (D). Values are presented as mean ± SD. *P < .05 vs control; #*P* < .05 vs low-dose; ∼*P* < .05 vs high-dose, ^*P* < .05 vs low-dose recovery.

Rohypnol, at low and high doses, significantly increased gastric MDA level when compared with the control. Interestingly, the increase in gastric MDA was significantly reversed to the control value following a drug-free recovery period. In addition, there was a significant fall in gastric GSH concentration in the rohypnol treated rats when compared with the control. The decrease in gastric GSH was noted to be dose-dependent. The observed fall in gastric GSH was partially reversed following drug withdrawal. Furthermore, rohypnol caused a dose-dependent rise in the gastric level of GSSG, but a decline in GSH/GSSG ratio, when compared with the control group. Rohypnol-induced alterations in GSSG and GSH/GSSG were reversible following cessation of rohypnol exposure (Figure 1).

There was a significant decrease in the SOD activities in the gastric tissue of the rohypnol-treated rats when compared with control. Although, the alterations in SOD activities were completely reversed to the control value in the low-dose rohypnol recovery group, the high-dose rohypnol animals showed partial reversal. Also, high-dose rohypnol treatment led to a significant irreversible decrease in catalase activities when compared to all other groups. In addition, rohypnol treatment caused a dose-dependent decline in GST activities in the gastric tissue when compared with control. The decline in GST activities was observed to be irreversible by rohypnol cessation. Furthermore, rohypnol treatment caused a significant irreversible decline in GPx activities in the gastric tissue compared with the control (Table 2).

**Table 2.** Effect of rohypnol on gastric enzymatic antioxidant activities.

Group/parameter	Control	Low-dose rohypnol	High-dose rohypnol	Low dose-rohypnol recovery	High dose-rohypnol recovery
Superoxide dismutase (U/mg)	156.6 ± 16.97	119.9 ± 9.63*	100.5 ± 13.5*	138.3 ± 11.2^∼^	125 ± 21.6*^∼^
Catalase (μmol/g)	6.04 ± 0.53	5.28 ± 0.67	4.27 ± 1.12*	4.96 ± 1.13	3.46 ± 1.15*^#^
Glutathione-S-transferase (U/mg)	5.53 ± 0.79	4 ± 0.55*	2.66 ± 0.35*#	4.16 ± 1.1*^∼^	3.2 ± 1.0*
Glutathione peroxidase (μmol/g)	6.51 ± 0.82	3.81 ± 0.76*	2.83 ± 0.60*	3.59 ± 1.43*	2.53 ± 0.95*

Notes: Values are presented as mean ± SD. *P < .05 vs control; ^#^*P* < .05 vs low-dose; ^∼^*P* < .05 vs high-dose.

### 3.3. Effect of rohypnol on markers of inflammation

**Figure 2.**
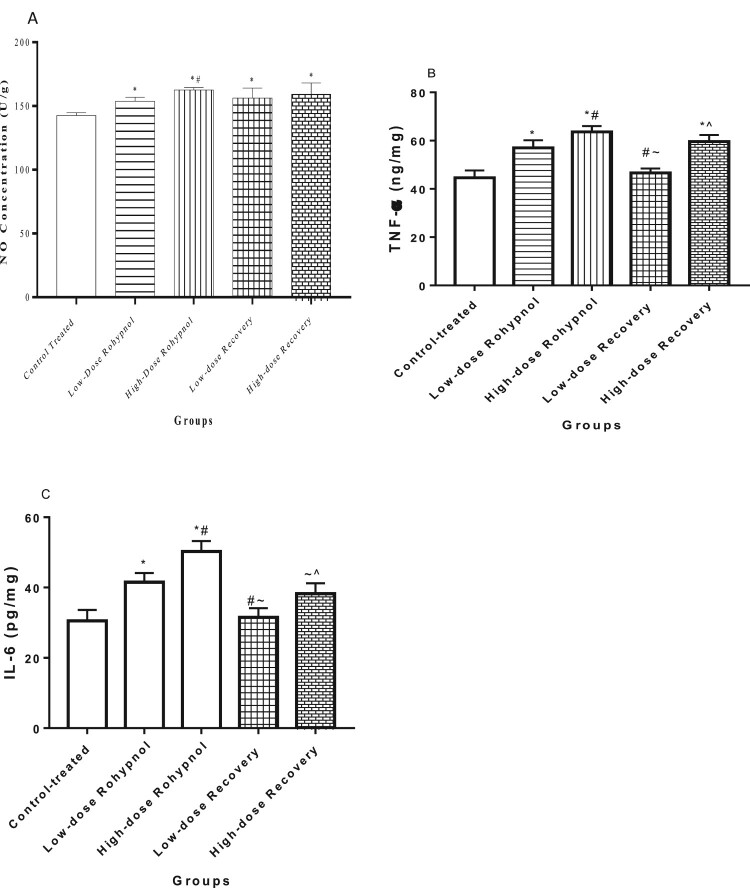
Effect of rohypnol on gastric levels of nitric oxide, NO, (A), tumor necrotic factor-α, TNF-α (B), and interleukin-6, IL-6 (IL-6) (C). Values are presented as mean ± SD. *P < .05 vs control; #*P* < .05 vs low-dose; ∼*P* < .05 vs high-dose, ^*P* < .05 vs low-dose recovery.

Rohypnol treatment caused a significant rise in NO concentration in the gastric tissue compared with control. The rise in gastric NO level was found to be dose-dependent and irreversible. In a similar way, rohypnol exposure caused a rise in gastric TNF-α and IL-6 concentrations when compared with the control animals. The rise in IL-6, but not TNF-α, was significantly reversed in high dose rohypnol-treated rats; however, the rise in TNF-α and IL-6 was significantly reversed in the low-dose rohypnol-treated animals (Figure 2).

**Figure 3.**
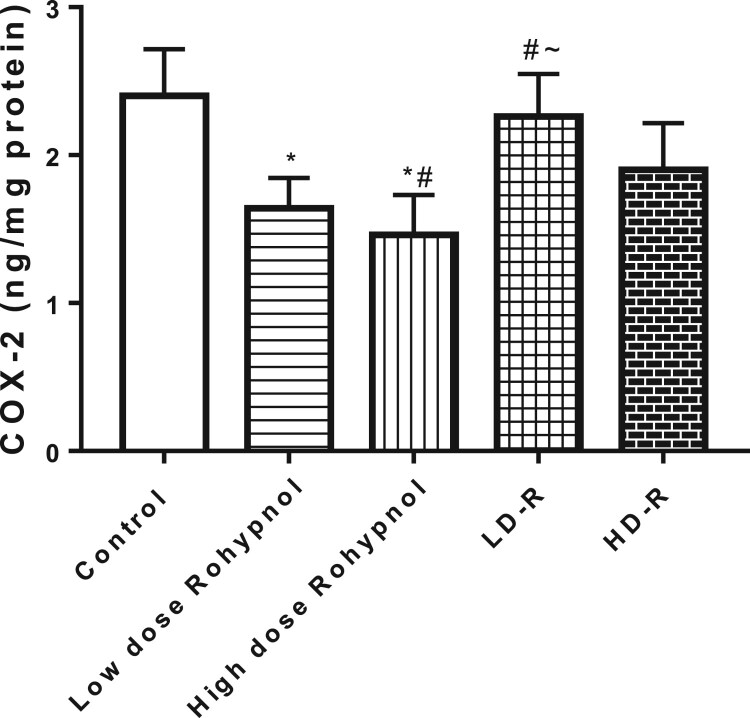
Effect of Rohypnol on gastric levels of cyclo-oxygenase-2 (COX-2). Values are presented as mean ± SD. *P < .05 vs control; #*P* < .05 vs low-dose; ∼*P* < .05 vs high-dose.

In addition, rohypnol led to a decrease in gastric concentration of COX-2 in a dose-dependent manner. This alteration was reversible in the low dose-treated rats but no in the high dose-treated rats (Figure 3).

### 3.4. Effect of rohypnol on markers of DNA damage and apoptosis

Rohypnol treatments led to a significant increase in 8-OHdG concentration in the gastric tissue when compared to control rats. When compared with the control, the rise in 8-OHdG was completely reversed in the low-dose rohypnol-treated rats but partially reversed in the high-dose rohypnol-treated rats. In addition, rohypnol caused a significant dose-dependent increase in gastric DFI. The observed rohypnol-induced rise in gastric DFI was irreversible. High dose but not low dose rohypnol treatment led to a rise in gastric caspase 3 activity compared with the control. The noted rise in caspase 3 activity was irreversible (Table 3).

**Table 3.** Effect of rohypnol on gastric genotoxicity and apoptotic markers.

Group/parameter	Control	Low-dose rohypnol	High-dose rohypnol	Low dose- rohypnol recovery	High dose- rohypnol recovery
8-OHdG (ng/mg)	0.49 ± 0.07	0.68 ± 0.07*	0.77 ± 0.12*	0.59 ± 0.37	0.69 ± 0.16*
Gastric DFI (%)	17 ± 3.30	36 ± 4.04*	47.75 ± 5.4*#	29.63 ± 8.2*^∼^	45.13 ± 2.95*#
Caspase-3 (ng/mg)	0.368 ± 0.22	0.561 ± 0.18	0.86 ± 0.09*	0.43 ± 0.26^∼^	0.77 ± 0.37*

Notes: Values are presented as mean ± SD. *P < .05 vs control; #*P* < .05 vs low-dose; ^∼^*P* < .05 vs high-dose.

### 3.5. Histopathological findings

**Figure 4.**
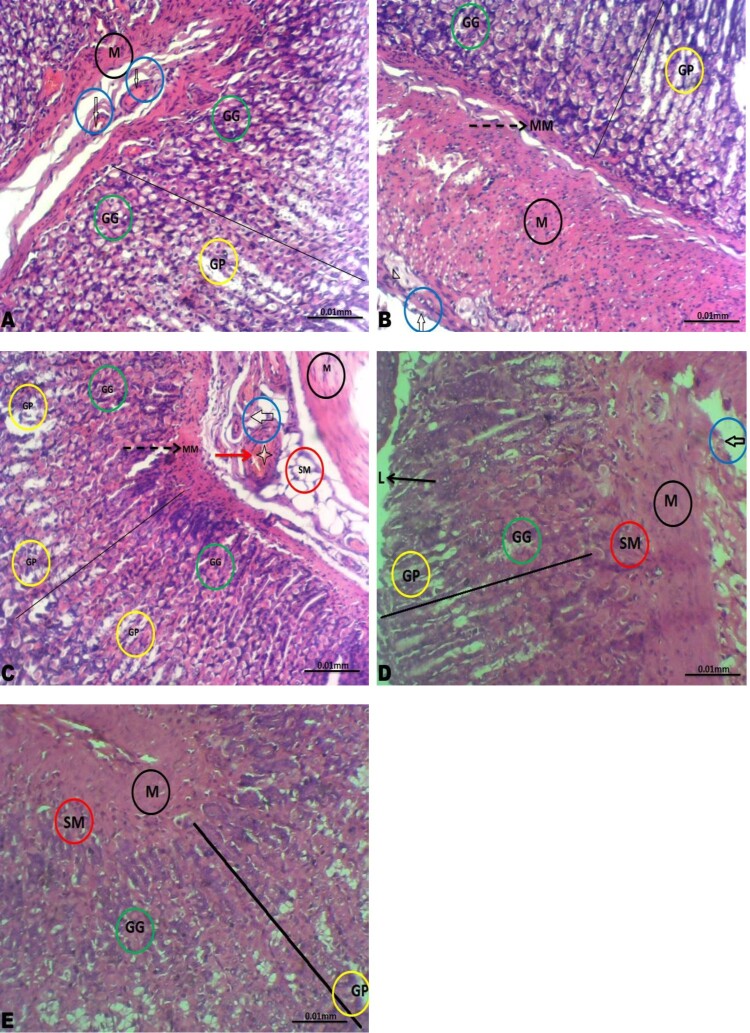
Photomicrographs of the gastric tissue. The gastric tissues of the control (A), low dose Rohypnol-treated (B), and the low dose recovery (D) and high dose recovery (E) groups showed preserved layers; the mucosa (line) lined by simple columnar epithelium lying on a thin layer of the muscularis mucosa (MM, broken black arrow), and the submucosa (SM in red circle) composed of branches of blood vessels (white arrow in blue circle) and lymphatics, the muscularis (M in black circle) and adventitia. The gastric glands (GG in green circle) and gastric pit (GP in yellow circle) appeared unremarkable. However, the gastric tissue of the high dose Rohypnol-treated (C) revealed hyperemic blood vessels (white arrow in blue circle) and moderately atrophied gastric gland (GG in green circle).

The gastric tissues of the control, low-dose rohypnol-treated, and the recovery groups showed preserved layers; the mucosa lined by simple columnar epithelium lying on a thin layer of the muscularis mucosa, and the submucosa composed of branches of blood vessels and lymphatics, the muscularis and adventitia. The gastric glands and gastric pit appeared unremarkable. However, the gastric blood vessels of the high-dose rohypnol-treated animals appeared hyperemic and the gastric glands appear moderately atrophied (Figure 4).

Furthermore, high dose rohypnol treatment led to significant gastric mucosa lesion index and pathological injury integral when compared with other groups. This was considerably reversible following cessation of rohypnol exposure (Table 4).

**Table 4.** Effect of rohypnol on gastric mucosa integrity.

Group/parameter	Control	Low-dose rohypnol	High-dose rohypnol	Low dose- rohypnol recovery	High dose- rohypnol recovery
Gastric mucosa lesion index	0.20 ± 0.44	0.60 ± 0.54	2.20 ± 0.45*^#^	0.40 ± 0.55^∼^	0.80 ± 0.83^∼^
Gastric mucosa pathological injury integral	0.25 ± 0.50	0.75 ± 0.50	3.00 ± 0.81*^#^	0.50 ± 0.57^∼^	0.75 ± 0.50^∼^

Notes: Values are presented as mean ± SD. *P < .05 vs control; #*P* < .05 vs low-dose; ^∼^*P* < 0.05 vs high-dose.

### 4. Discussion

Rohypnol toxicity has emerged as an important global concern following increased abuse. Like most drugs of abuse, its exposure prevalence and attendant toxic effects makes it a predisposing risk factor for organ toxicity. Hence, understanding the impacts and associated mechanisms of rohypnol exposure is of utmost significance in the maintenance of the health of exposed individuals and those at risk of exposure. The results of this study could be used in policy making and health awareness with regards to rohypnol abuse. Findings of the present study provide insights into the toxic effects of rohypnol exposure on the stomach, while depicting the possible ameliorating role of rohypnol cessation and oxidative-sensitive mechanisms, such as glutathione system-dependent redox state and caspase 3-mediated apoptosis, involved in the pathogenesis.

Oxidative stress denotes an excessive generation of reactive oxygen species (ROS) beyond the buffering capacity of the antioxidant system [21]. The glutathione system is essential in enhancing the buffering capacity of the antioxidant system and maintaining cellular redox balance [13–15]. The role of oxidative stress and glutathione system in drug-induced organ damage is well documented [19,23,33]. In this study, we observed substantial evidence of oxidative stress induction in the gastric tissue of rats treated with rohypnol. There was a significant rise in MDA, GSSG, and NO concentrations, which was accompanied by suppression of non-enzymatic and enzymatic antioxidants such as GSH (and GSH/GSSG), SOD and catalase as well as GST and GPx in rohypnol-exposed rats. This adds up to the existing literature that associate drugs of abuse with depletion of the antioxidant systems, including glutathione system, thus promoting ROS accumulation and oxidative injury [33].

Additional evidence that starts the induction of oxidative stress was the observation of increased gastric NO content. NO is produced from the guanidine group of L-arginine by nitric oxide synthases (NOS). The inducible form of NOS, iNOS, is activated by proinflammatory agents like IL-1β, TNF-α, and interferon-γ [34]. Reports in the literature have shown that superoxide reacts with NO to generate peroxynitrite, a highly reactive oxidant [35], that promotes oxidative injury and inflammation [19,36]. Although iNOS and peroxynitrite were not assayed in the present study, findings from this study may suggest that the rohypnol-induced oxidative stress may be intricately linked to the rise in proinflammatory cytokines (TNF-α and IL-6), which may possibly activated iNOS and led to increased gastric NO. Although there are no available reports in the literature to compare our present findings on rohypnol-induced NO rise, previous studies have documented the potentials of drugs, especially when abused, to trigger NO increase in tissues [19,31,36]. Thus, it follows that NO rise as observed in the current study may promote gastric oxidative and inflammatory injury in response to enhanced proinflammatory cytokines.

COX-2 is a product of an immediate response gene in inflammatory cells and it is induced by endotoxins, mitogens, or cytokines, including TNF-α and IL-6 [37,38]. Hence, the findings of this study suggest that the observed downregulation of COX-2 is independent of rohypnol-induced upregulation of cytokines. It is likely that rohypnol exerts a negative regulatory effect on gastric mucosa integrity by downregulating COX-2, thus inhibiting the release of the gastroprotective prostaglandins [39,40] and exposing the gastric mucosa to oxidative injury.

The mechanisms outlined above possibly explain the histopathological observations in this study as well as the increased gastric mucosal lesion index and gastric mucosa pathological injury integral. From our findings, the lesions attributable to rohypnol toxicity include vascular hyperemia and atrophy of the gastric glands, which is possibly a consequence of oxido-inflammatory injury triggered by rohypnol exposure. It is a known fact that oxidative stress and inflammation increases membrane permeability [31], which promotes vascular congestion. This leads to proton pump dysfunction, apoptosis, and DNA damage [31]. These findings were coupled with reduced gastric weight in rohypnol-treated rats, particularly those treated with high dose, compared to the control, irrespective of a sparing effect noticed on the body weight. The reduced absolute and relative gastric weight in response to rohypnol treatment is likely a reflection of the rohypnol-induced oxido-inflammatory injury and gastric gland atrophy, and an indicator of the toxic effect of rohypnol [41].

Data obtained from the various parameters evaluated in the present study consistently revealed the protective potential of rohypnol cessation after some period of exposure. Although withdrawal did not completely reverse rohypnol-induced damage, it significantly ameliorated the observed rohypnol-induced perturbations. Rohypnol withdrawal after four weeks of exposure significantly blunted rohypnol-induced rise in gastric MDA, GSSG, NO, TNF-α, and IL-6 while also stimulating a rise in GSH, and activities of SOD, catalase, GST, GPx, and COX-2. These observations were associated with downregulation of gastric caspase 3 activity and reduced gastric DNA damage evidenced by reduced gastric 8OHdG and DFI following rohypnol withdrawal. Furthermore, the rohypnol withdrawal led to restoration of gastric cytoarchitecture and weight. These findings may infer that rohypnol-induced gastric injury is reversible, although studies with prolonged exposure (beyond four weeks as used in this study) may be required to validate this claim.

In conclusion, the data obtained from this study revealed that rohypnol induced gastric injury via an redox-sensitive mechanism by suppressing gastric glutathione content and COX-2 activity, and upregulating caspase 3-dependent apoptosis. Also, it is worthy to note that rohypnol-induced gastric damage was not completely, but partially, reversed by rohypnol withdrawal after exposure.

## Ethical approval

The Ethics Committee of Oyo State Ministry of Health, Ibadan, approved the experimental protocol (reference number: AD/13/479/1786A).

## Data Availability

The data that support the findings of this study are available on request from the corresponding author.
